# The instrumental value of advance directives: lesson learned from the COVID-19 pandemic for policymaking

**DOI:** 10.1007/s43999-025-00060-6

**Published:** 2025-02-05

**Authors:** Elisabeth Stock, Christian H. Nickel, Bernice S. Elger, Andrea Martani

**Affiliations:** 1 Institute for Biomedical Ethics, University of Basel, Basel, Switzerland; 2 Emergency Center, University Hospital Basel, Basel, Switzerland; 3 Center for Legal Medicine, University of Geneva, Geneva, Switzerland

**Keywords:** Advance Directives, Advance Care Planning, Resource allocation, COVID-19 pandemic, Instrumental value

## Abstract

Open conversations between patients and healthcare professionals (HCP) are required to evaluate which treatments are reasonable for the individual case, especially towards the end of life. Advance Care Planning (ACP), which often results in drafting an Advance Directive (AD), is a useful tool to help with decisions in these circumstances, but the rate of AD completion remains low. During the COVID-19 pandemic, ACP and AD gained popularity due to the alleged advantage that they could facilitate resource allocation, to the benefit of public health. In this article, which presents a theoretical reflection grounded in scientific evidence, we underline an even stronger ethical argument to support the implementation of AD in end-of-life care (eol-C) i.e. the instrumental value at the individual level. We show, with particular reference to lessons learned from the COVID-19 pandemic, that AD are instrumentally valuable in that they: (1) allow to thematise death; (2) ensure that overtreatment is avoided; (3) enable to better respect the wish of people to die at their preferred place; (4) help revive the “lost skill” of prognostication. We thus conclude that these arguments speak for promoting the territorially uniform implementation and accessibility of high-quality AD in care.

## Introduction

The rapid pace of medical innovation has led to substantial improvements in quality of life, but it also raises questions about the appropriateness of new medical interventions. Indeed, medical progress is accompanied also by increases in the risk of over-diagnosis and overtreatment [1]. In this context, providing good quality care requires open conversations between individual patients and their HCP to evaluate which treatments are reasonable, especially towards the end of life [2–4]. And yet, as documented by a report of the Organisation for Economic Co-operation and Development (OECD), professionals are frequently at unease to start conversations about options that ensure a dignified end-of-life, and there is a lack of documentation of individuals’ care preferences. Further, end-of-life care (eol-C) does not always achieve the aim of alleviating suffering and limiting unnecessary treatments sufficiently [5–10].

Two useful means have been developed to help improving eol-C: Advance Care Planning (ACP) and Advanced Directives (AD). An AD “*is a statement made in advance of an illness about the type and extent of treatment one would want*,* on the assumption that one may be incapable of participating in decision-making about treatment when the need arises*” [11]. In other words, AD is a tool to assert patients’ autonomy, especially in delicate end-of-life situations. Drafting AD often happens as a result of ACP, which is the terminology generally used to describe structured procedures that HCP set up to help patients reflect on “*personal values*,* wishes*,* expectations and ideas regarding treatment and care in the event of illness*,* accidents or the need for medical attention*” [12]. Although the two terms differ (AD emphasises the result, ACP the process), they both refer to the elaboration of indications on treatment preferences for a future where the patients may not be able to express their choices. For this reason, we will use the term AD throughout the article to refer to both, unless specified.

The importance of AD to help improve eol-C has been accentuated by the COVID-19 pandemic. Healthcare systems were faced with resource scarcity and it became more relevant to know which patients desired– or not – which treatment. The idea of a fair allocation of resources in the pandemic often included a call to consider patients’ wishes to encourage “*all patients*,* especially those facing the prospect of intensive care*,* to document in an advance care directive what future quality of life they would regard as acceptable and when they would refuse ventilators or other life-sustaining interventions can be appropriate*” [13]. Indeed, although evidence on the connection between AD and better resource allocation is lacking– as we highlight later on– during the pandemic it was common to argue that “*A thorough approach to obtaining advance directives might alleviate some of the burden on scarce resources*” [14].

In this article, which presents a theoretical reflection grounded in scientific evidence, we aim to explore the role of AD as a tool to improve eol-C in light of resource scarcity from an ethical perspective. Specifically, we seek to emphasize the instrumental value of AD at the individual level. Although AD in the pandemic were increasingly debated as a potential means to help allocate resources between different patients, hospitals and geographical areas, we underline their value as a means to reach other valuable ends related to the provision of good eol-C. The initial section of this article sketches a brief history of AD and the challenges to their implementation in eol-C, using Switzerland as a case example. Subsequently, we discuss the impact of the COVID-19 pandemic on AD, their implementation and prominence in policy debates. We show there has been a shift in that AD have been increasingly framed as a public health tool. Finally, we argue against this vision and show that, from an ethical perspective, future policymaking on AD should rather focus on their *instrumental* value to improve eol-C at the individual level, in particular, to help: thematise death, avoid overtreatment, better respect the wish of people to die at their preferred place and revive the “lost skill” of prognostication.

## Short overview of traditional challenges in AD implementation with Switzerland as a case example

Implementing AD in eol-C has traditionally been difficult, since this concept is not very old. In Switzerland, for example, it has only been present for a few decades. In the 1980s, end-of-life therapeutic decisions were mostly made by physicians, often with the objective of biological life prolongation. Only thereafter the topic of overtreatment became hotly debated, and a desire for more autonomy by patients, especially in the context of severe illness, became prominent. The primary focus was on implementing self-determination as a personal right to decline medical procedures even when patients would become unable to explicitly express their wishes (e.g. due to being unconscious). The first model for AD was introduced in Switzerland in 1984 upon initiative of a private organisation that also provides assisted suicide [15]. Although some local policies on AD came into place in the decades after [16], no extensive national legislation existed until 2013, when new rules explicitly regulated AD and their legal validity. Nevertheless, AD have few formal requirements and a current reform strives to offer a harmonised implementation without unjustified regional differences [17]. The core aim to improve ACP and implement AD is to improve eol-C [18].

Although in Switzerland– like in many other countries– AD now are increasingly accepted as useful tool to improve eol-C, many challenges remain. These are related to their accurate, territorially widespread and ethically sound implementation. For a start, there is a substantial geographical variation in terms of the pre-printed forms, which are used to collect patients’ wishes and then turn them into legally-binding AD. The shortest forms offer space for patients to appoint a surrogate decision maker, while others include the opportunity to determine future medical treatments and/or also contain the opportunity to make statements of personal values [19]. Some forms are available in all official languages and thus can be used throughout the country. In addition, many institutions offer their own forms. This results in the availability of different forms nationally and locally. The fact that these forms differ content-wise becomes clear when comparing two pre-printed forms from the Italian-speaking and German-speaking parts of Switzerland. The first– targeting residents of a certain nursing home– also include a call to update or renew it at least bi-annually and contains a question about antibiotics [20]. The latter was created and published in cooperation with different local organisations and includes a question about the motivation for making an AD and offers space to express thoughts on life and death [21].

Substantial regional variation in terms of the forms used for AD is an issue common to many countries, such as the United States, as an analysis of AD documents from different states revealed [22]. On top of the absence of standardised forms, the literature identified additional problems that hinder the implementation of AD. These include the issue that pre-printed forms “*are too hypothetical to cover all important aspects and therefore offer space for misunderstandings and misinterpretation*” [19]. Other authors cast doubts on whether AD can really help protect patient autonomy [23], since it is difficult to make accurate future-oriented decisions.

## AD and the COVID-19 pandemic: a changing tide?

Implementing AD has traditionally been a challenge, as suggested by the theoretical considerations exposed above. Furthermore, research has demonstrated that rates of AD completion differ across countries, although overall completion levels remain notably low [24–26]. In Switzerland, a 2018 study found that only 24% of persons aged 55 + completed an AD and that this percentage varied significantly between German-speaking (28.7%), French-speaking (10.3%), and Italian-speaking regions (17.9%) [56]. Similar percentages were also found in the US, as before the pandemic about 25% of adults had an AD, with a range of 10–71%, the percentage raising by the age of the persons [27].

However, AD gained popularity during the COVID-19 pandemic [28, 29], and it seems this marked an important change of tide for the implementation of these tools. Possible reasons are that there was an increase in hospitalisation rates and deaths due to COVID-19 and many older people living with frailty faced such risks [30–32]. Completing an AD *after* hospital admission was difficult because the rapid and severe progress of the illness made it impossible for many patients to decide about treatment themselves [27]. Accomplishing an AD *before* a critical illness occurred became therefore crucial to avoid that the individual wishes of patients were not accurately documented. There were many calls in the scientific literature to promote and increase the use of AD due to the pandemic. Block et al. wrote that “[…] *we see an urgent need to prepare older adults and other at-risk populations for the possibility of severe illness through a massive upscaling of ACP […]*” [33]. Curtis et al. reasoned that ACP should be of high priority and that physicians should provide life-sustaining treatment only to patients who explicitly want them, acting according to patients` goals and values. They deemed this crucial to avoid futile or undesired high-intensity care when the healthcare capacity is at its limits [30].

Despite the promotion of AD during the COVID-19 pandemic, it is not always easy to determine whether uptake increased. In Switzerland, a greater awareness about the importance of conversations about serious illness developed and a focus on the documentation of the will of patients was emphasized [34]. As part of a mixed-methods project our team is conducting [35], preliminary results based on interviews with HCP suggest that sometimes people were encouraged to make AD on initiatives of primary care physicians and that in some nursing homes people were asked to fill in (or revise) AD to add information about their preferred treatment in case of COVID-19. Older persons also showed greater interest in filling in an AD and more often consulted their primary care physicians regarding AD. These preliminary results match some reports in the grey literature. For example, a Swiss newspaper article suggested that HCP in leading positions fostered people to fill in AD, and that a non-profit organisation helping people to manage their eol-C witnessed a high increase in AD uptake [36].

There are also published studies showing an increased prominence of AD, particularly in the first months of the pandemic. Auriemma et al. [37] monitored users of an online tool for ACP, and found that AD monthly completion rates increased from pre-COVID-19 to the COVID-19 period. Possible explanations included the heightened awareness of AD among the public due to COVID-related hospital visitation limitations, the increased encouragement of physicians to promote ACP and patients finding a new motivation to complete AD due to the pandemic [37]. In Colorado, a retrospective observational study on user rates of an online based ACP tool showed that “*over a 5-month period that includes the early phase of the COVID-19 pandemic in Colorado*,* total monthly use of the advance care planning portal tool increased from 418 users in January to 1037 users in April*” [38]. Similar results were found in West Virginia: Although there was only a rise of 9.05% within the first six months of AD forms submitted to an official nationwide registry, the number of DNR cards submitted increased by 77,18%. This increase rate may reflect patients’ concerns, who did not want maximal medical efforts at the end-of-life if infected by COVID-19 [28]. Dassel et al. also showed increasing awareness of AD during the COVID-19 pandemic: 63% of the surveyed Caregivers stated that their care recipient had an AD and since the outbreak of the pandemic 19% revised their document [39].

## AD during resource scarcity: a useful tool for better resource allocation?

As we discussed above, implementing AD has traditionally been difficult, but since the COVID-19 pandemic there are signs that AD gained popularity. This renewed interest for AD sparked a broad discussion on the ethical considerations related to their implementation in contexts of resource scarcity. Various problems exist to capture patient wishes adequately and challenges to ensure high quality AD increased: During the early months of the COVID-19 pandemic, the understanding of the virus and knowledge about treatment options was limited [40]. Modifying AD based on such limited knowledge raises ethical concerns, as individuals may struggle to express wishes about illnesses they are not well-acquainted with. This emphasizes Fagerlin et al.`s [23] critique of AD who highlight the challenge in foreseeing future desires. Moreover, overload of information from the media that may have reinforced the impression that older individuals were unequivocally vulnerable, with only few ‘survivors’ [41, 42]. It is not known to what extent such narratives may have impacted in the content of AD, e.g. in relation to the willingness to forgo treatment. An analysis of English-language newspaper articles on COVID-19 and AD showed that they often emphasised the “*focus continued to be on treatment and care options […] and [was] less likely […] to include information on preparation for medical decision making by discussing values and goals*” [43]. We are not aware of any studies that investigated the impact of media coverage of COVID-19 on AD uptake and content in different regions, but there is a report from Taiwan were authors speculate that the increase of AD completion was due to media reports depicting the gravity of the situation [44]. Further, there is a tendency that older adults show a higher level of altruism than younger individuals. This became crucial especially during the COVID-19 pandemic when people waved treatment on altruistic motives [45, 46]. When intentions of treatment preferences are not carefully evaluated with trained HCP during the AD filling process it might result in low-quality AD. This bears the danger of injustice and there are indications that this disproportionately impacts women [47, 48].

Despite these considerations, many policies and recommendations considered that a further implementation of AD during a situation of resource scarcity like the COVID-19 is helpful. It was argued that AD, although being originally conceived as tools to promote individual welfare by empowering patients to obtain the desired eol-C, could actually be used also to promote collective welfare, by favouring a better allocation of scarce resources. In Switzerland, the COVID-19 task force established by the Federal Government emphasised that *“The primary goal of [AD] in the context of COVID-19 is to avoid unwanted hospitalizations and intensive care treatment*,* which would unnecessarily burden these health care provisions and aggravate the need for rationing”* [49]. Similarly, in the ethical literature it was advanced that “*advance care planning is a means of alleviating some uncertainty around end-of-life care preferences and should be utilized for the ethically guided use of limited resources*” [50]. In brief, the argument was proposed that AD are a useful means to use for allocation of scarce resources between different regions, hospitals and patients. It implies a partial shift in the focus of AD, which turn from a tool primarily aimed at promoting individual welfare, to one centred around societal welfare, public health and resource allocation. This was also suggested by the American Geriatrics Society, who– although admitting that AD “*are not rationing and should not be confused with triage allocation decisions*”– also claimed that AD discussions “*are of paramount importance to reduce the need to ration limited healthcare resources during an emergency because these discussions will identify people who do not wish to receive intensive care including mechanical ventilation*” [51]. Should therefore a more prominent focus on the public health value of AD guide future discussions and policymaking in the area of eol-C?

The question whether AD can really be a good means to achieve more justly and ethically allocation of scarce resources between different patients, hospitals and geographical areas, remains open. Indeed, there are no studies to our knowledge that investigate the impact of AD on resource allocation during the COVID-19 pandemic. In absence of empirical evidence, it thus seems premature to focus on the public health value of AD. Moreover, it was argued that the emphasis on the public health value of AD can be dangerous, since older people “*may also feel pressured to forego intensive care ‘voluntarily’ […] due to the prominence that age has gained both in public discussions of limiting access to intensive care*” [52]. Finally, whilst we acknowledge that different discourses on the function of AD may co-exist, too much emphasis on the alleged (but unverified) usefulness of AD as a public health tool to improve resource allocation risks to overshadow another side of AD that is particularly important in situations of resource scarcity. As we show in the next section, the renewed centrality that AD have assumed in policymaking during the COVID-19 pandemic is a chance to reinstate their *instrumental* value in promoting individual welfare.

### AD’s *instrumental * value to promote individual welfare

In the previous sections, we showed that AD were brought to the centre of attention during the pandemic. We then argued that in policy discourse around resource allocation, AD were sometimes framed as a useful tool to improve distribution of scarce resources from the public health perspective. This is problematic not only for the reasons listed above, but also because it overshadows the *instrumental* value AD have in respect to promoting individual welfare. In ethics, something has an instrumental value when it does not possess worth in itself, but because it is conductive to something of final value [53]. The renewed emphasis on AD during the pandemic should thus not be exploited to emphasise the importance that these tools do have ‘in and of themselves’, but as a means to achieve other final values that are relevant for policymaking in eol-C and which focuses on individual-level care, rather than public health. In the following paragraphs we expose four different facets of the *instrumental* value of AD. We postulate, that this *instrumental* value requires high-quality AD including in-depth discussions led by trained HCP with patients to foster their self-determination and autonomy during the AD filling process with a minimum standard of appointing a therapeutic representative.

### AD as an instrument to thematise death

The topics of dying and death remains a taboo in many societies [54–57]. Death is frequently not encountered first hand for long periods of people’s lives, e.g., by keeping children away from dying people and funerals [55]. The lack of exposure to death leads to feelings of helplessness when confronted with this topic. Medical and technical progress raises the hope of a constant prolongation of life, a high percentage of patients die in a very old age and death and dying have become institutionalized [55].

According to a survey conducted in the UK during the COVID-19 pandemic with members of the public, a majority of respondent felt that society does not talk enough about death and dying. Only 14% expressed their future health and care preferences in advance about severe illness and dying [58]. In some cases, HCP do not consider the process of death and dying as their responsibility. This becomes evident when thinking of the well-known phrase *nothing can be done any more* showing that in these cases there is a focus on curative competence [59]. Indeed, death and dying as topics are rarely a central part in clinical everyday life and medical education, despite recommendations to the contrary. The Swiss Academy of Medical Sciences suggests in medical ethics guidelines that it is important to communicate to patients with a deadly disease the seriousness of a prognosis and a high likelihood of dying, in an early stage [60].

Addressing the topic of death can be demanding for both patients and HCP. Avoiding conversations about death and dying bears the danger of inappropriate treatment at the end of life [61]. This points at one important *instrumental* value that AD can have. Since ACP discussions usually consider especially the end-of-life period, they encourage individuals and HCP to bring up the topic of death and determine how to go about it [34, 57, 62]. This highlights the relevance of integrating death education for HCP and the public in future policies on eol-C, as underlined by a recent call for action [27] and following recommendations given in a report of the Lancet Comission on the Value of Death: “*Education on death*,* dying*,* and end-of-life care for a person and their family must be integral*,* substantial*,* and mandatory in the curriculum of every health and social care student and continuing education for practicing professionals*” [61].

### AD as an instrument to avoid overtreatment

According to a study conducted in Germany, the “*use of intensive care services during terminal hospitalizations increased across all age groups*,* particularly the elderly*” [63]. Hartog et al. doubted, that intensive care in end-of-life stages of older people is also according to their will [64]. Furthermore, they depict that many physicians, nurses, and ambulance workers frequently perceive certain treatments as over-therapy. The wishes of the patient play a key role in this context. Uncertainty about the patients’ preferences and doubts about overtreatment put healthcare professionals into a stressful position, especially when they are confronted with resource scarcity. The authors make a call for action that more effort needs to be done to know the patients’ will before an acute situation occurs and suggest ACP interventions [64]. Also Gupta et al. see a high urgency to increase the knowledge about patients’ preferences since “*Patients whose wishes are left unsaid often receive burdensome life sustain therapy by default*,* prolonging patient suffering*” [65]. In most western health care systems, a focus on curative interventions is the primary approach in medical treatment and HCP may be pressured to provide treatments to hospitalized patients, because through these interventions this system is financed [66]. Nevertheless, patients’ preferences should be the guiding factor to determine which medical procedures they desire or omit. Hence, the principle of autonomy, particularly the patient’s right to make independent decisions regarding their own healthcare, is among the most important in medical ethics [67].

In this context, AD can function as valuable instruments in preventing overtreatment. In fact, when healthcare professionals are made aware of patients’ explicit preferences, they are compelled to align their actions with patients’ autonomy. The potential AD have in achieving the final aim of avoiding overtreatment at the individual level is also confirmed by some research. In a recent German retrospective analysis of health insurance data, a decrease in curative overtreatment at the end-of-life (e.g. new percutaneous endoscopic gastrostomy for some at the very end of life) was partly explained by political initiatives that emphasised the importance of AD [68]. Similarly, a famous Australian randomised-controlled trial on ACP showed that they help in avoiding unwanted life-prolonging treatment [69].

### AD as an instrument to better respect the wish of people to die at their preferred place

The preferred place of dying and the actual place of death varies between different countries. In Germany, numerous people in need of care live at home and many of them express the wish to die at home, but a very common place of death is the hospital [70]. In a representative study conducted 2017 Switzerland, ~ 72% expressed the wish to die at home, but about 80% die in hospitals and nursing homes [71, 72]. According to an US survey (*n* = 2847 individuals age 65 or older) the majority of the respondents express a preference for spending their final days at home in the case of a terminal illness with less than a year to live [73]. In Canada, a national study has shown *“Home is not universally the preferred setting for dying. The public*,* especially older persons and those expressing lower expectations of families in general*,* express greater preference for palliative care settings […]”* [74]. In England and Wales most people prefer to die at home, but only ~ 20% of individuals aged 65 or older and ~ 10% of persons older than 85 years do so [75]. One reason for unwanted hospitalisation in end-of-life stages is when the preferred place of death has not been discussed in advance [76]. Driller et al. found in their cohort study “*that ACP conversations in primary health care is a method to support the wish of many patients to stay at home as long as possible and to get the opportunity to die at home*” [77].

This shows how AD hold a significant *instrumental* value to allow people realise their wish to die in their preferred place. If individuals communicate their preferred place of dying through AD it enhances the likelihood of their desires being honoured, particularly if expressed early on. There is indeed some evidence, albeit limited, that AD help patients to die in the preferred place [78]. AD can then empower both family members and the care team to coordinate essential measures, such as arranging home-based palliative care. This alleviates the burden on family as it ensures preparedness for this end-of-life phase, facilitating the establishment of necessary arrangements.

### AD as an instrument to revive the “lost skill” of prognostication

Physicians often hesitate to discuss the likely prognosis with their patients even though many of them would agree that conversations about expected outcomes are important. In a qualitative study, physicians frequently considered patients’ prognosis without directly conversing about it with their patients, and had differing opinions on whether and when a discussion about long-term prognosis is necessary. Further, they assessed life expectancy rather with “clinical gestalt” instead of using validated tools, which lead to a wide variation of prognostication time frames [79]. The reasons why physicians avoid conversations about prognosis, however, are poorly understood. A study on Code Status discussions in the hospital setting (i.e. patients’ preferences in the case of a cardiopulmonary arrest), showed that about 60% of patients did not remember having had such a discussion even though a Code Status was documented in a vast majority of cases in the electronic health record [80]. Such deficiencies in communication have been already demonstrated 30 years ago [81]. A systematic review shows that specific communication interventions appear to be associated with lower preference for life-sustaining therapies if patients had received such a communication intervention [82]. These results suggest that well-informed patients (through such interventions) could potentially enhance participation in decision-making. The likely prognosis is important for both treatment decisions as well as goals of care discussions [83]. Primary care physicians, for example, are uniquely positioned to help patients understand how prognosis will affect their likelihood of benefitting or being harmed by medical interventions or therapies. In the primary care sector, patients frequently have longstanding relationships with their patients which is helpful for conducting such conversations [84]. AD serve as an *instrumental* value that will foster such conversations and can contribute– in a way– to revive the “lost skill” of prognostication [85].

## Conclusion

The COVID-19 pandemic has accentuated the importance of AD and brought them back to the forefront of societal and scientific discussions. Future policymaking should not, however, emphasise the newly developed focus of AD as a public health tool to help with resource allocation, since further research is required to find out whether AD can really be a good means to achieve a more just and ethical allocation of scarce resources between different patients, hospitals, and geographical areas. It is much more important that policies highlight the *instrumental* value that ACP and AD can have to help reach four goals at the individual level: thematising death, reducing overtreatment, help patients die at their preferred place, and revive the “lost skill” of prognostication. Achieving these goals requires ACP and AD of high-quality led by trained HCP to foster patients` self-determination and autonomy to avoid the risk of AD implicated injustice such as altruistic motivated treatmentwaiver. This can be an ethically solid way of exploiting the hype around ACP and AD stimulated by the pandemic, without however changing the focus of these tool from the individual to the societal level.

## Data Availability

Not applicable.
